# Genetic diversity of *Plasmodium vivax *in Kolkata, India

**DOI:** 10.1186/1475-2875-5-71

**Published:** 2006-08-14

**Authors:** Jung-Ryong Kim, Mallika Imwong, Amitabha Nandy, Kesinee Chotivanich, Apichart Nontprasert, Naowarat Tonomsing, Ardhendu Maji, Manjulika Addy, Nick PJ Day, Nicholas J White, Sasithon Pukrittayakamee

**Affiliations:** 1 Department of Clinical Tropical Medicine, Faculty of Tropical Medicine, Mahidol University, Bangkok, Thailand; 2 Department of Parasitology and Protozoology, the Calcutta School of Tropical Medicine, Kolkata, India; 3 Centre for Clinical Vaccinology and Tropical Medicine, Churchill Hospital, Old Road, Headington, Oxfordshire OX3 7LJ, UK; 4 The Royal Institute of Thailand, Grand Palace, Bangkok, Thailand

## Abstract

**Background:**

*Plasmodium vivax *malaria accounts for approximately 60% of malaria cases in Kolkata, India. There has been limited information on the genotypic polymorphism of *P. vivax *in this malaria endemic area. Three highly polymorphic and single copy genes were selected for a study of genetic diversity in Kolkata strains.

**Methods:**

Blood from 151 patients with *P. vivax *infection diagnosed in Kolkata between April 2003 and September 2004 was genotyped at three polymorphic loci: the *P. vivax *circumsporozoite protein (*pvcs*), the merozoite surface protein 1 (*pvmsp*1) and the merozoite surface protein 3-alpha (*pvmsp*3-alpha).

**Results:**

Analysis of these three genetic markers revealed that *P. vivax *populations in Kolkata are highly diverse. A large number of distinguishable alleles were found from three genetic markers: 11 for *pvcs*, 35 for *pvmsp*1 and 37 for *pvmsp*3-alpha. These were, in general, randomly distributed amongst the isolates. Among the 151 isolates, 142 unique genotypes were detected the commonest genotype at a frequency of less than 2% (3/151). The overall rate of mixed genotype infections was 10.6%.

**Conclusion:**

These results indicate that the *P. vivax *parasite population is highly diverse in Kolkata, despite the low level of transmission. The genotyping protocols used in this study may be useful for differentiating re-infection from relapse and recrudescence in studies assessing of malarial drug efficacy in vivax malaria.

## Background

Malaria remains one of the most important communicable diseases in the world. Despite enormous control efforts over many decades malaria is still a significant health problem. It is estimated that around 300–500 million cases occur each year with one to three million deaths. The problem is compounded by multiple drug resistance in *Plasmodium falciparum *and chloroquine resistance in *Plasmodium vivax *[1]. The global burden of malaria due to *P. vivax *is 70–80 million cases annually. Vivax malaria is usually a non-lethal infection but its prolonged and recurrent infection can have major deleterious effects on personal well-being, growth and on the economic performance at the individual, family, community and national levels [2]. The recent emergence of chloroquine-resistant strains is of great concern [3-5].

*P. vivax *causes about 60–65% of all malaria infections in India [6,7,42]. The frequency of relapse after a standard course of chloroquine and primaquine treatment is 23–40% depending on the duration of follow-up in India [7,36]. *P. vivax *and *P. falciparum *are prevalent in all age groups but their prevalence is highly seasonal and differs between the species; longitudinal studies in India show a winter peak for *P. falciparum *and a summer peak for *P. vivax *[7,8]. Chloroquine appears to remain an effective drug in the treatment of *P. vivax *malaria in Kolkata [6].

The majority of studies on the genetic structure of *Plasmodium *have focused on *P. falciparum*, using polymorphic markers such as the merozoite surface protein-1(*msp*-1), -2 (*msp*-2), glutamate-rich protein (*glurp*) [9,14]. A similar approach has been adopted for *P. vivax *but it has been less well-studied at the molecular level than *P. falciparum *[18]. Three polymorphic *P. vivax *genes have been widely used for molecular epidemiological studies. The *pvcs *gene has a central repeat domain that varies in sequence and number of repeat units [10,41]. Two major types, VK 210 and VK247, have a worldwide distribution and four subtypes from VK210 and two subtypes from VK 247 can be differentiated by restricted enzyme digestion to show polymorphisms in both the pre- and post- repeat region [28]. The *pvmsp*1 gene has been used to determine whether an infection is a result of a new infection or a relapse [11] and used to genotype isolates of different strains from different geographical regions [12,13,15-17]. The polymorphic *pvmsp*3-alpha gene was also studied by polymerase chain reaction-restriction fragment length polymorphism (PCR-RFLP) analysis [19,20]. The *pvmsp*3-alpha gene encodes a merozoite surface protein with an alanine-rich central domain that is predicted to form a coiled-coil tertiary structure [21]. There is added interest in the *pvmsp3 *antigen family as immunogens and vaccine candidates. Use of the *pvmsp*3-alpha gene as genetic marker has been recently validated [19,20,24,30,39,40]. Other genetic markers used for *P. vivax *are the apical membrane antigen 1 (Ama-1), gametocyte antigen 1 (*gam*1) and *msp*3-beta. The PvAma-1 is a protein essential for erythrocyte invasion, which shows limited sequence polymorphism [26,34]. The potential of *pvgam*1 as a molecular marker for genotyping is compromised by artifacts associated with amplification of this region [29]. The *pvmsp*3-beta gene is a member of a family of related merozoite surface proteins containing a central alanine-rich central region with significant genetic diversity [22,30]. Despite this high level of sequence diversity certain physical properties of the encoded protein are maintained, particularly the ability to form coiled-coil tertiary structure [22], which may limit genetic studies.

Studies using single or combined *pvcs*, *pvmsp*1 and *pvmsp*3-alpha genotyping have assessed the genetic diversity of *P. vivax *isolates from various regions. A study on *pvcs *identified that the VK247 genotype was widely distributed and was the predominant form in Thai and Papua New Guinea isolates but its prevalence was much lower in Mexico [23]. Another study, by contrast, revealed that the VK210 type in dimorphic *pvcs *gene was found in the majority of the parasites in Thai strains [24,10]. A recent single gene study of *pvmsp*3-alpha revealed that it was highly polymorphic, and that three major types of the *pvmsp*3-alpha locus could be distinguished [32,39]. Moreover earlier studies revealed a high prevalence of multiple genotype infection as determined by *pvmsp*3-alpha [19] and *pvcs *genotyping [18]. Recently a combined *pvcs *and *pvmsp*1 study showed a lower rate of multiple genotype infections than an earlier study and high polymorphism in Thai strains [28]. In hyperendemic areas, intragenic recombination and high genetic diversity have been reported in *pvmsp*1 and *pvmsp*3-alpha [16,20,39]. Even in hypoendemic areas, such as Thailand and Brazil [11], *pvmsp*1 and *pvmsp*3-alpha display high levels of diversity [24]. By contrast, relatively low genetic diversity of *pvmsp*1 has been detected in the re-emerging vivax malaria focus in Korea [31]. Little is known about the genetic diversity among parasite populations in India, where most vivax malaria in the world occurs. Earlier studies carried out using isoenzyme typing was consistent with the random mating nature of vivax malaria isolates in India [27]. Recent studies on the polymorphism of *pvcs*, *pvgam*1 and *pvmsp*3 alpha in Indian isolates have revealed two types of *pvcs *and nine size variations of *pvgam*1 and high polymorphism in the *pvmsp*3 alpha gene [37]. Therefore, the highly polymorphic, single-copy, unlinked genes, *pvcs*, *pvmsp*1 and *pvmsp*3-alpha were selected for this study of genetic diversity of *P. vivax *in Kolkata.

## Methods

### Blood samples

Venous blood samples were collected from patients with symptomatic *P*. *vivax *malaria (n = 151) attending the malaria clinic in the Calcutta School of Tropical Medicine, India, between April 2003 and September 2004. All patients gave fully informed consent to participate in this study which was approved by the Ethics committee of the Faculty of Tropical Medicine, Mahidol University and also permitted by the Calcutta School of Tropical Medicine. The diagnosis of vivax malaria was made by examination of thin and thick blood smear. Patients with pregnancy, G6PD deficiency and any underlying haemolytic diseases, and aged below three years old were excluded. Chloroquine (25 mg base/kg) either alone or in conjunction with a five- or fourteen-day regimen of primaquine (15 mg base/day), was given to the 151 patients; primaquine was given only to non-G6PD deficiency cases. G6PD tests were done at the Centre for Tropical Medicine and Parasitology. Blood samples were collected in EDTA tubes from patients with confirmed *P*. *vivax *infection and were stored at -20°C until DNA extraction.

### DNA template preparation

DNA was purified from 1 ml of infected venous blood using a commercially available DNA extraction kit (QIAGEN, Germany) according to a procedure described previously [28].

### Amplification protocol

A nested PCR approach was adopted following previously described procedures [28,19]. All amplification reactions were carried out in a total volume of 20 μL in the presence of 10 mM Tris-HCl, pH8.3, 50 mM KCl, 250 nM of each oligonucleotide primer, 125 μM of each of the four dNTPs and 0.4 units of ampli-Taq polymerase (Invitrogen, USA). Primary amplification reactions were initiated with 1 μL of the template genomic DNA prepared from the blood samples, and the 1 μL of the product of these reactions was used to initiate the secondary amplification reaction. The PCR parameters were as follows: an initial denaturation step at 95°C for five minutes preceded the cycles of annealing at a temperature defined for each primer pair for two minutes, extension at 72°C for two minutes, and denaturation at 94°C for one minute. After a final annealing step followed by five minutes of extension, the reaction was terminated by reducing products temperature to 25°C. PCR products were stored at 4°C until analysis.

### Analysis of the amplification product

The DNA fragments obtained from PCR were analyzed by electrophoresis in agarose gels. For direct analysis of the fragments, 10 μL of the amplified PCR product were applied to 1.5% horizontal agarose gels for *pvcs*, 2.0% for the second fragment of *pvmsp*1 and 1.0% for *pvmsp*3-alpha at 120 volts. The size of the amplified DNA was estimated by comparing its mobility to a molecular weight marker. A 100 bp DNA ladder was used as a molecular weight marker in gel electrophoresis for *pvcs *and the second fragment of *pvmsp*1 but a 1 kb DNA ladder (Life and Technologies, U.S.A) was used as a molecular weight marker in the gel electrophoresis for *pvmsp*3-alpha as this contains a 1900 bp DNA fragment. For restriction fragment length polymorphism analysis of the PCR products (PCR-RFLP), 10 μL of the amplified PCR product were first digested with a restriction enzyme (New England Biolabs Inc, UK) for three hours in a total volume of 20 μL, before applying to 1.5% or 1.8% agarose gels. Electrophoresis was performed in TBE buffer, and the DNA was visualized on an ultraviolet transilluminator following ethidium bromide staining. For the purpose of this study, the frequency of an allele is calculated simply as its percentage of the total of all alleles detected among the isolates examined.

## Results

### Genotyping of *pvcs*

**Figure 1:**
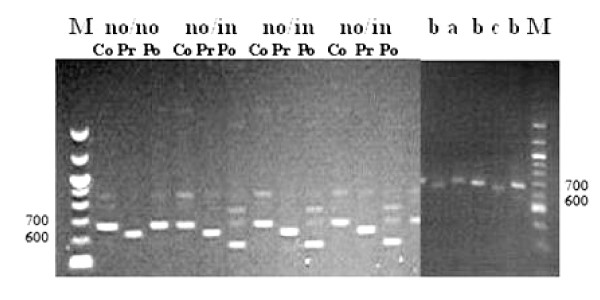
The predominant two variants (no/no and no/in) of VK210 type of pvcs gene observed in the 150 isolates from Kolkata, India. M is a 100 bp DNA ladder. Co and Pr and Po stand for control (nested PCR product), pre-repeat region digested by *ScrF1 *enzyme and post-repeat region digested by *Bbs1 *enzyme, respectively. The three size variants **a**, **b **and **c **are 740, 710 and 680 bp, respectively.

Amplification of the fragment containing the *pvcs *gene was attempted for blood samples obtained from patients. After two rounds of PCR, bands of 680–740 bp were amplified. This nested PCR generated three product size variants (Table 1). All the allelic variants were ordered in decreasing sizes of base pairs and coded alphabetically, starting **a **to **c**. The frequencies of type **a **(740 bp), type **b **(710 bp) and type **c **(680 bp) were 28%, 65% and 7%, respectively (Figure 1). The most common allele was type **b **(65%; 97/151). Two cases of type **a **were mixed with type **b**. There were two types of repeat region in the *pvcs *gene: the VK210 type of repeat unit coding for Gly-Asp-Arg-Ala-Asp/Ala-Gly-Gln-Pro-Ala [41], and the VK247 type coding for repeats of Ala-Asn-Gly-Ala-Gly-Asn-Gln-Pro-Gly [10]. VK210 and VK247 were distinguished by digestion with the restriction enzymes, *Alu*1 and *Bst*N1. In 151 samples 150 isolates were VK210 type and only one isolate was VK247 (Table 1).

**Table 1:** Size and RFLP patterns and populations of *pvcs *alleles (n = 151).

Alleles	Size variation	Pre/Post patterns	n (%)
1. VK247	a	in	1 (0.66)
2. VK210	a	in/in	2 (1.32%)
3. VK210	a	no/in	26 (17.2%)
4. VK210	a	no/no	16 (10.6%)
5. VK210	a+b	no/in	2 (1.32%)
6. VK210	b	in/in	4 (2.64%)
7. VK210	b	no/in	48 (31.7%)
8. VK210	b	no/no	45 (29.8%)
9. VK210	c	in/in	1 (0.66%)
10. VK210	c	no/in	2 (1.32%)
11. VK210	c	no/no	5 (3.3%)

**Figure 2:**
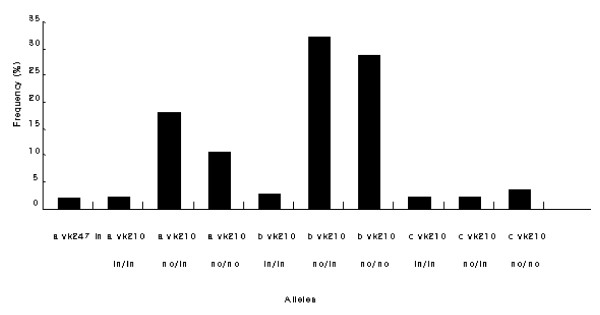
Allele frequency distribution of *pvcs *observed in the 151 Kolkata isolates.

PCR products were digested by *Scr*F1, which recognizes insertion on 5'CC/NGG3' restriction site in the pre repeated region and by *Bbs*1, which cuts the DNA on 5'GAAGACNN/NN3' site in post repeated region. Therefore, there are four possible combinations in the VK210 type (no/in, no/no, in/in and in/no) but in VK247 type two types were possible (in or no). Three variants were found in the samples (no/no, no/in, and in/in) and two of them (no/no and no/in) were predominant in the Kolkata strains (Figure 1). The frequencies of these were 43.7% for no/no and 51% for no/in respectively. Eleven alleles of *pvcs *(combinations of size variation and insertion presence/absence) were found in 151 isolates, and these were randomly distributed (Figure 2).

### Genotyping of *pvmsp*1

The second fragment of *pvmsp*1 generated 1087–1150 bp from 151 samples.

**Figure 3:**
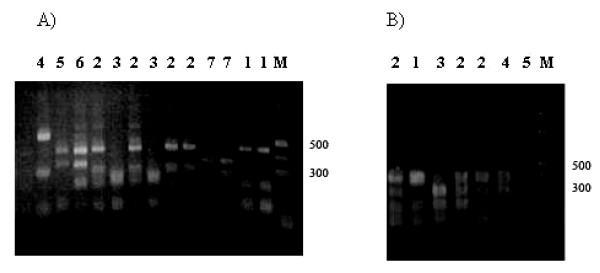
A) Dominant alleles by *Alu1 *restriction enzyme digestion of the second fragment of *pvmsp1 *gene. B) Dominant alleles by *Mnl1 *restriction enzyme digestion of the second fragment of *pvmsp1 *gene. M is a DNA marker in 100 bp steps.

Analysis on agarose gels revealed two different sizes, labeled **a **and **b**. Despite the lack of variation in the size of the amplification products, RFLP analysis with *Alu*1 and *Mnl*1 restriction enzymes revealed substantial diversity at the nucleotide level. The RFLP patterns of different isolates showed variation in size. Digestion with either *Alu*1 or *Mnl*1 yielded fragment sizes that were highly polymorphic. From the 151 samples 8 different *Alu*1 patterns and 5 different *Mnl*1 patterns were observed (Figure 3). When data from both analyses were combined, 35 alleles of the second fragment of *pvmsp*1 could be differentiated (Table 2).

**Table 2:** Size and RFLP patterns and frequency of *pvmsp1 *alleles detected (the second fragment) (n = 151).

Alleles	Size variation	*Alu*1 RFLP patterns	*Mnl*1 RFLP patterns	n (%)
1	a	a1	m1	20 (13.2%)
2	a	a1	m2	2 (1.32%)
3	a	a2	m1	3 (2.0%)
4	a	a2	m2	17 (11.2%)
5	a	a2	m4	1 (0.66%)
6	a	a2	m5	1
7	a	a3	m1	1
8	a	a3	m6	1
9	a	a4	m1	1
10	a	a4	m2	1
11	a	a4	m4	1
12	a	a5	m4	6 (4.0%)
13	a	a5	m5	1
14	a	a6	m4	2
15	a	a6	m5	13 (8.6%)
16	a	a7	m4	1
17	a	a7	m5	1
18	b	a1	m1	26 (17.3%)
19	b	a1	m2	5 (3.3%)
20	b	a2	m1	1
21	b	a2	m2	7 (4.6%)
22	b	a3	m1	1
23	b	a3	m2	1
24	b	a3	m5	1
25	b	a4	m1	2
26	b	a5	m1	1
27	b	a5	m2	2
28	b	a5	m4	16 (10.3%)
29	b	a6	m1	1
30	b	a6	m2	2
31	b	a6	m4	4 (2.64%)
32	b	a6	m5	2
33	b	a7	m4	1
34	b	a7	m6	4
35	b	a8	m5	1

**Figure 4:**
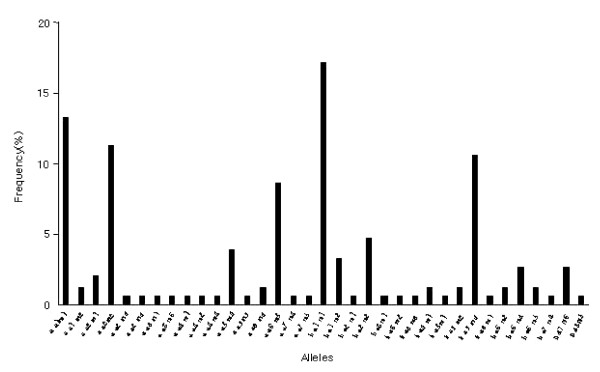
The allele frequency distribution of the second fragment of *pvmsp1 *locus in the 151 Kolkata isolates characterized by a combination of fragment size and sequence type using RFLP.

The frequency distribution of the 35 alleles of the second fragment of *pvmsp*1 is shown in Figure 4. It was found that alleles 1 (**a **a1 m1) and 18 (**b **a1 m1), alleles 4 (**a **a2 m2) and 28 (**b **a5 m4) were particularly predominant (20 and 26, 17 and 16 respectively among 151 samples [13.2, 17.2, 11.2 and 10.6% respectively]). The remaining isolates were randomly distributed between the other variants.

### Genotyping of *pvmsp*3-alpha

**Figure 5:**
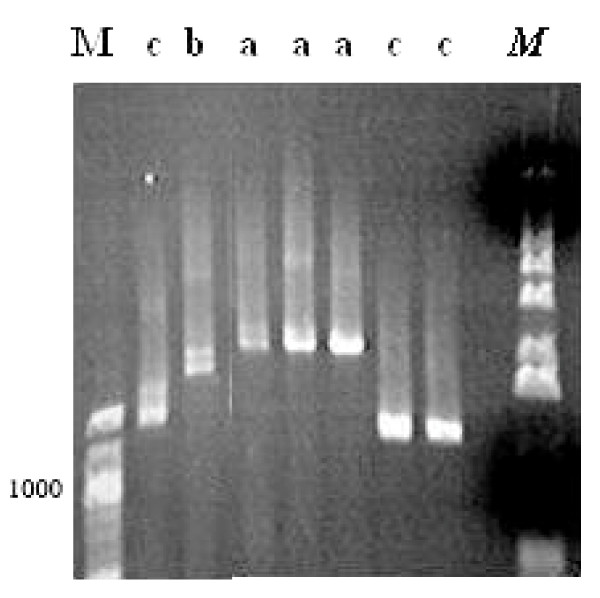
Three sizes of *pvmsp3*-alpha gene product in the 151 Kolkata isolates. The three sizes **a**, **b **and **c **represented are 1900, 1500 and 1100 bp, respectively. M is a 100 bp DNA ladder marker and *M *is a 1 kb DNA ladder. Double bands reflect infection with multiple genotypes.

**Figure 6:**
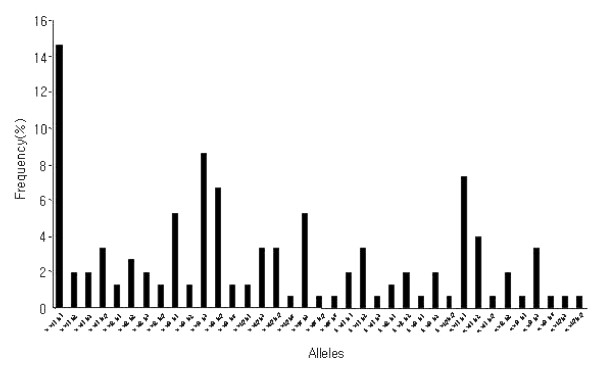
Frequency distribution of alleles at the *pvmsp3-*alpha loci observed in the 151 Kolkata isolates.

Amplification products showed a major size polymorphism with three different variants labelled **a**, **b **and **c **(1900-1100 bp). These were predominantly of **a **size (102/151 [67.5%]) (Figure 5). The frequencies of type **a **(1900 bp), type **b **(1500 bp) and type **c **(1100 bp) were 67.5%, 12.5% and 20% respectively. RFLP analysis with *Alu*1 and *Hha*1 restriction enzymes revealed substantial diversity at nucleotide level. Digestion with these enzymes yielded fragment sizes that were highly polymorphic. The sum of the fragment sizes did not always equal the size of the intact PCR product, indicating non-resolvable variation in size of the uncut amplification products but never more than the total size, thereby indicating a number of mixed genotypes. From the 151 samples, five different *Alu*1 patterns and five different *Hha*1 RFLP patterns were observed. When data from both analyses were combined, 37 alleles of *pvmsp*3-alphacould be differentiated (Table 3). The frequency distribution of the 37 alleles of *pvmsp*3-alpha alleles in 151 isolates is shown in Figure 6. Allele 1 (**a **al1 h1) was particularly dominant (22/151 [14.5%]) and the others were randomly distributed.

**Table 3:** Size and RFLP patterns and frequency of *pvmsp*3-alpha alleles detected (n = 151).

Alleles	Size variation	*Alu *1 RFLP patterns	*Hha*1 RFLP patterns	n (%)
1	a	al 1	h1	22 (14.5%)
2	a	al 1	h2	3
3	a	al 1	h3	3
4	a	al 1	h4	5 (3.3%)
5	a	al 2	h1	2
6	a	al 2	h2	4
7	a	al 2	h3	3
8	a	al 2	h4	2
9	a	al 3	h1	8 (5.2%)
10	a	al 3	h2	2
11	a	al 3	h3	13 (8.6%)
12	a	al 3	h4	10 (6.6%)
13	a	al 3	h5	2
14	a	al 4	h1	2
15	a	al 4	h3	5
16	a	al 4	h4	5
17	a	al 4	h5	1
18	a	al 5	h3	8 (5.2%)
19	a	al 5	h4	1
20	a	al 5	h5	1
21	b	al 1	h1	3
22	b	al 1	h2	5
23	b	al 1	h3	1
24	b	al 2	h1	2
25	b	al 2	h2	3
26	b	al 3	h1	1
27	b	al 3	h3	3
28	b	al 4	h4	1
29	c	al 1	h1	11 (7.2%)
30	c	al 1	h2	6 (4.0%)
31	c	al 1	h4	1
32	c	al 2	h2	3
33	c	al 3	h1	1
34	c	al 3	h3	5
35	c	al 3	h5	1
36	c	al 4	h3	1
37	c	al 4	h4	1

### Analysis of three genes genotyping

The results showed marked genetic diversity in the Kolkata strains with 11 alleles for *pvcs*, 35 alleles of the second fragment of *pvmsp*1, and 37 alleles of *pvmsp*3-alpha. Mixed genotypes of each marker were found in *pvcs *1.3%, *pvmsp*1 0.7% and *pvmsp*3-alpha 8.6%. The overall rate of mixed gene infection was 10.6% using all three genetic markers. The most common alleles for each of the three genes were seen in 31.7% of samples for *pvcs *(allele7-**b**VK210 no/in), 17% of samples for the second fragment of *pvmsp*1 (allele18-**b **a1 m1) and 14.5% for *pvmsp*3 (allele1-**a **a11 ha1) (Figure 2, 4, and 6). Therefore, when genetically homogeneous parasite lines are considered in this area, the three markers can distinguish a potential 14,245 (11 × 35 × 37) unique genotypes. In the combination of three genetic markers, The most common three marker genotype was **b **VK210 no/in, **b **a1 m1, **c **a1 h1 allele, which occurred at a rate of 2% (n = 3/151).

## Discussion

The results showed that *P. vivax *parasites from Kolkata demonstrated an extremely high prevalence of VK210 type in the *pvcs *gene (with only one VK247 type), high polymorphism in both merozoite surface proteins (*pvmsp*1 and *pvmsp*3 alpha), and low rates (10.6%) of multiple genotype infection.

The predominance in Kolkata isolates of VK210 *pvcs *gene type (99.3%) has not been seen elsewhere to the same extent. Recent studies in Thailand have found rates of 70.5% [35], 78% [24] and 90% [28], though these results were in sharp contrast to those of earlier studies in which VK247 was found to be the predominant type; 83% in Thai and 90% in Papua New Guinea samples by PCR/Oligo Probe [23]. Another study of Indian strains has shown the VK210 type to be predominant, but also demonstrated (using a different technique) significant numbers of VK247 type [33]. This phenomenon may be attributed to selection by host immune pressure on a particular genotype, and/or the preferential production of sporozoites carrying a specific variant [18], such as VK210 for *pvcs *in a mosquito species. These differences may also be due to sampling biases or regional temporal fluctuations of individual genotypes frequencies. The frequency of absence of the pre-repeat insertion of VK210 in the *pvcs *gene was 4.7% (7/150) and that of the post-repeat region of the gene 56% (84/150) in Kolkata. In the absence of a multicentre study involving various regions of India it is difficult to define the extent to which the observed difference was in any way related to the functional aspects of the Kolkata strain.

In this study, two major types of the *pvmsp*1 marker containing 35 alleles were found. A similar degree of diversity (36 allelic types) was also found in the study from Thailand using the same laboratory protocol [28], and with a different protocol in Papua New Guinea and Indian strains [16,42]. For *pvmsp*3-alpha, the present study reveals 37 alleles with three size variants cut by two restriction enzymes. The frequencies of the three *pvmsp*3-alpha types were consistent with those found in Papua New Guinea and Thailand [18,19]. A recent study using a different protocol revealed 16 size and sequence polymorphic allele in Indian strains [37]. This high degree of polymorphism in the merozoite surface protein (*pvmsp*1 and *pvmsp*3-alpha) is similar to that found in other studies from other regions.

All three markers show marked genetic diversity in Kolkata strains, with 11 alleles for *pvcs*, 35 alleles for the second fragment of *pvmsp*1, and 37 alleles for *pvmsp*3-alpha. This compares with another study of Indian vivax isolates which revealed 2 sequence variants of *pvcs*, 9 size variants in *pvgam*1, and 16 size variants with sequence polymorphism in *pvmsp*3-alpha gene [37].

The frequency of multiple genotype infections of *P. vivax *malaria has been estimated in many regions but it is difficult to compare these values due to differences in sampling and genotyping methods. The proportion of mixed gene infections estimated in Papua New Guinea, India, and Thailand ranges from 30% to 65% [16,20,24,25]. The present study with a relatively large sampling size, shows an overall 10.6% for multiple genotypes (1.3% for *pvcs*, 0.7% for *pvmsp*1 and 8.6% for *pvmsp*3 alpha). This rate may reflect a limitation in the sensitivity of PCR for the detection of multiple genotype infections, despite the high degree of polymorphism seen.

According to the Calcutta School of Tropical Medicine Report 2005, the malaria clinic treats around 6,000 malaria positive cases annually, of which an average of 65% are caused by *P. vivax*. The rate of mixed infection with both *P. vivax *and *P. falciparum *during the last few years has declined (1.0% in 1997, 0.7% in 1998 and 0.1% in 2001) [6]. The samples from Kolkata were collected from an urban area where vivax malaria remains chloroquine-sensitive, but where recurrences are frequent. Only a few cases were contracted outside of central Kolkata. The high degree of polymorphism and low level of multiple genotype infection probably reflects the nature of this endemic setting. More study is needed to assess whether these discrepancies reflect true differences between disease populations, or are due to differences in sample sizes or the laboratory methodology [20,38].

## Conclusion

When genotyped using three polymorphic markers (*pvcs*, *pvmsp*1 and *pvmsp*3-alpha)*P. vivax *infections in Kolkata demonstrate a low rate of multiple genotype infection despite a high degree of genetic diversity. Given the degree of diversity found genotyping methods such as these are likely to be of value in distinguishing relapse/recurrence from reinfection in clinical trials of antimalarial drug efficacy.

## Authors' contributions

JRK was involved in all stages of this study. SP and AN designed the study and were responsible for the day-to-day supervision of patient recruitment and clinical management. MI was responsible for the supervision of the molecular genetic study and NT for the laboratory work. AM and MA participated in the recruiting of patients. KC and APN participated in the coordination of laboratory work. NJW and NPD helped compose the manuscript and gave constructive advice. All authors read and approved this final manuscript.
